# Perceived Coach-Created Empowering and Disempowering Climate Effects on Athletes’ Intentions to Use Doping: The Mediational Role of Self-Regulatory Efficacy and Attitudes towards Doping

**DOI:** 10.3390/sports12040100

**Published:** 2024-04-01

**Authors:** Beatrice Hoppen, Saulius Sukys

**Affiliations:** Department of Physical and Social Education, Lithuanian Sports University, Sporto 6, LT-44221 Kaunas, Lithuania; beatrice.hoppen@lsu.lt

**Keywords:** empowering climate, disempowering climate, doping likelihood, attitudes, self-regulatory efficacy, athletes

## Abstract

Background: The purpose of this study was to examine the relationship between perceived coach-created empowering and disempowering motivational climate and doping likelihood among athletes and whether the attitudes towards doping and doping self-regulatory efficacy mediates these relationships. Methods: Athletes (N = 948; 50% male; mean age, 20.32, SD = 2.45) recruited from a variety of sports completed questionnaires assessing their perceptions of coach-created motivational climate, attitudes towards doping, doping self-regulatory efficacy, and doping likelihood. Results: The study’s results showed significant negative direct effects of a perceived empowering climate on doping likelihood (β = −0.50) and attitudes towards doping (β = −0.48), and a positive effect on self-regulatory efficacy (β = 0.48). On the contrary, a disempowering climate had positive direct effects on doping likelihood (β = 0.53) and attitudes towards doping (β = 0.45), and a negative effect on self-regulatory efficacy (β = −0.49). Significant indirect effects on doping likelihood via attitudes and self-regulatory efficacy were found. Conclusions: Our findings suggest that athletes who perceive a more empowering climate created by the coach are less likely to use banned substances due to their more negative attitudes towards doping and stronger belief in their ability to resist the temptation to use doping.

## 1. Introduction

Cheating in sports could be best described as the use of illegal performance enhancing drugs or methods. Athletes that cheat in sports usually break the rules to gain some benefit [1], such as wastes of time or the use of prohibited substances. Doping is the use of a banned substance or method that could be harmful to an athlete’s health or has the potential to improve his/her performance in sport [2]. Doping is a massive problem in sports, as the latest research suggested that as many as 57.1% of elite athletes are using banned substances [3]. Gleaves et al. [4] reported that doping prevalence rates in competitive sports were up to 73%. Another study stated that 58% of elite athletes reported an interest in doping [5]. These numbers are much higher than 0.65% of athletes with a positive doping test [6]. Therefore, it is necessary to understand why athletes use doping, so that the prevalence of doping could be reduced. 

Some factors may explain why athletes cheat in sports by using doping. One such factor includes attitudes towards doping. It was found that attitudes towards doping were positively related to cheating behavior [7]. A review of studies related to doping in sports has shown a moderate effect of attitudes on doping use [8]. Another review confirmed that attitudes influence and predict doping susceptibility and doping behavior among competitive athletes [9]. More recent studies also displayed a positive relationship between doping attitudes and doping susceptibility [10] and doping intentions [11]. Therefore, some studies suggest that both attitudes and intentions are some of the most important psychological factors that can indicate doping behavior in sports [12,13]. 

Another important psychosocial process that could enhance our comprehension of doping in sports is self-regulatory efficacy [8]. According to Bandura [14], self-regulatory efficacy is believing in the capability to withstand both individual and social pressures to act in a harmful way. This is learned and maintained by favorable individual experiences, as well as by the influence of people around us. For example, athletes who associate themselves with popular role models of famous athletes who refrain from using doping are also more likely to believe in their ability to resist the use of doping [15]. A research study by Ntoumanis et al. [8] discovered that self-efficacy had a strong negative association with the intention to dope and doping behavior. More recent studies conducted by Ring and Kavussanu [15] with college athletes participating in both team and individual sports revealed that athletes who have high doping self-regulatory efficacy are not prone to doping. Another study, whose aim was to examine whether doping likelihood varies between benefit and cost situations, found that doping self-regulatory efficacy is negatively associated with doping likelihood [16]. It is important to mention that self-regulatory efficacy acts as a mediator in predicting doping intention among adolescent [17] and adult athletes [18]. 

Athletes take part in a social context by interacting with others. Therefore, an important factor that can influence athletes’ intentions to use doping is the motivational climate created by the coach. Coaches have a very important role in attaining athletic excellence [19,20] because they are in charge of creating a particular type of motivational climate when training, and it can affect how athletes deal with the tasks in sports [21]. The motivational climate is defined as a psychological atmosphere created by the coach [22]. Theoretical frameworks used in studies of motivation are the Achievement Goal Theory [23] and the Self-Determination Theory [24]. Both theories help to examine the intrapersonal motivational consequences of the social environment in sports. Based on the Achievement Goal Theory, a task-involving climate can be defined by various situations where the coach encourages athletes to improve skills, promotes individual progress, and focuses on cooperation with other athletes, so that every single athlete has a meaningful role in the team. On the contrary, an ego-involving climate focuses on victory, promotes competition with other athletes, and stimulates the social comparison between athletes [23]. On the other hand, the Self-Determination Theory explains two interpersonal styles defined as autonomy-support and controlling style [24]. An autonomy-support style focuses on athletes’ involvement in the decision-making procedure and athletes’ preferences. A controlling style uses controlling language, promotes coaches to force their opinion on athletes, pressures athletes in an authoritarian way, and controls the athletes’ personal lives [25]. The combined motivational climate characteristics from both theories proposed that the motivational climates encouraged by coaches are empowering and disempowering [26]. An empowering motivational climate can be defined by encouraging task involvement, boosting athletes’ autonomy, and providing social support. In contrast, a disempowering motivational climate could be best described as promoting both ego involvement and a controlling style.

A review by Birr et al. [27] found that an empowering motivational climate has beneficial effects on various psychological dimensions of athletes. It should be noted that studies have revealed associations between motivational climate styles and athletes’ moral behavior [28,29,30], as well as doping likelihood [31]. Therefore, to date, just one study was conducted to investigate the associations between coach-created empowering and disempowering climate and young soccer players’ predisposition to cheating [32]. For that reason, we are lacking scientific evidence on the association between coach-created empowering and disempowering climates and athletes’ doping likelihood.

The research described above suggests that attitudes towards doping and doping self-regulatory efficacy have been associated with doping likelihood. Moreover, a coach-created motivational climate could prevent athletes from doping by acting on the two factors mentioned above, namely attitudes towards doping and self-regulatory efficacy. It is reasonable to expect that athletes who perceive a more empowering climate should express a greater negative attitude towards doping, as well as feel more able to resist the use of doping. Indeed, a more empowering climate had a positive relationship with athletes’ self-regulatory efficacy [33,34] and also with attitudes towards doping [35]. However, no study has investigated whether attitudes towards doping and doping self-regulatory efficacy mediate the relationship between perceived empowering and disempowering climate and doping likelihood.

To fill this knowledge gap, the aim of the research was to investigate the relationship between perceived coach-created empowering and disempowering motivational climate and doping likelihood in a diverse sample of athletes and whether the attitudes towards doping and doping self-regulatory efficacy mediates these relationships. We hypothesized that perceived empowering motivational climate would be negatively correlated with doping likelihood and that this association would be mediated by attitudes towards doping and doping self-regulatory efficacy. We also hypothesized that a perceived disempowering motivational climate would have a positive relationship with doping likelihood and also mediated by attitudes and self-regulatory efficacy.

## 2. Materials and Methods

### 2.1. Participants

The participants of the current research were 948 athletes from Lithuania, who ranged in age from 16 to 37 years (M = 20.32, SD = 2.45). Half of the participants were male and competed in different individual (64.3%, e.g., track-and-field, swimming, gymnastics, and shooting) and team (35.7%, e.g., basketball, football, and handball) sports. At the moment of data collection, participants’ experience in their current sport ranged from 2 to 16 years (M = 8.44, SD = 2.63). Inclusion criteria stipulated that the participants had at least one year of sport experience.

### 2.2. Measures

#### 2.2.1. Empowering and Disempowering Motivational Climate

Athletes’ perceptions of coach-created empowering and disempowering characteristics of the motivational climate were measured by the Empowering and Disempowering Motivational Climate Questionnaire—Coach [36] adapted to the Lithuanian context [37]. The questionnaire consisted of 17 empowering items (e.g., “My coach acknowledged players who tried hard”, and “My coach gave players choices and options”) and 17 disempowering (e.g., “My coach had his or her favorite players”, and “My coach was less accepting of players if they disappointed him or her”) items. Athletes were instructed to “Think about how things have gone in your team most of the time during the last 3 or 4 weeks”. A five-point Likert-type scale, ranging from strongly disagree (1) to strongly agree (5), is used to answer the items. Higher scores reflect more characteristic motivational climates. Previous studies have shown good internal consistency of the empowering (α = 0.86) and disempowering subscales (α = 0.89) [37].

#### 2.2.2. Doping Self-Regulatory Efficacy

The Sport-Specific Doping Self-Regulatory Efficacy Scale [15,38] adapted to the Lithuanian context [39] was used to measure athletes’ perceived ability to resist doping. Participants had to express how confident they were in their ability to refrain from using illegal substances to enhance performance in sport by evaluating seven items (e.g., “When most athletes in your sport use them”, and “When pressured to do so by others”) by using a 7-point scale, ranging from 1 (*not at all confident*) to 7 (*completely confident*). Higher scores reflect a stronger athlete’s perceived ability to refrain from using illegal substances. Previous studies have shown that the reliability of this scale was very good (α = 0.96) [39].

#### 2.2.3. Attitudes towards Doping

The Performance Enhancement Attitude Scale [40] was used in order to measure athletes’ attitudes towards doping. More specifically, this scale has been validated for the Lithuanian context [41] and has shown that the shortened 8-item version has better psychometric properties. Using a Likert scale that varied from 1 (strongly disagree) to 6 (strongly agree), participants were required to indicate how much they agreed with each of the statements. A higher score reflects a more positive attitude towards doping. The prior studies have provided evidence for very good internal consistency of this scale (α = 0.93) [41].

#### 2.2.4. Doping Likelihood

Athletes’ likelihood to use doping was assessed by using two hypothetical situations [42], which were also used in research with Lithuanian athletes [41]. The first situation portrayed a setting where the participants could use a prohibited substance to improve performance, while the second portrayed a setting where the prohibited substance could be used to heal from injury. After reading these situations, participants had to indicate the likelihood to use the prohibited substances on Likert scale ranging from 1 (not at all likely) to 7 (very likely). A higher score indicated that the participants are more likely to dope. The good internal consistency of this combined measure was identified (α = 0.81) [42].

### 2.3. Procedures

After obtaining ethical approval from the Lithuanian Sports University, athletes were recruited from various sports teams and clubs in Lithuania. Before data collection, all participants provided informed consent. Participants were informed about the study’s aim; instructed about the voluntary nature of their participation with the option to withdraw at any point; and assured of strict confidentiality regarding data, which would be solely used for research purposes. After participants indicated their consent, they completed the questionnaire without the coach present. During this process, participants were provided with the researchers’ contact information for any inquiries. Questionnaires were administered to participants in classroom settings, with clear instructions that researchers would be available to address any questions they may have had. Additionally, one of the researchers remained present with participants throughout the questionnaire sessions to ensure smooth progress and address any immediate concerns. The response rate in this study was 84%.

### 2.4. Statistical Analysis

First, preliminary analyses were conducted to look for missing values and check data normality, followed by a reliability analysis, computed descriptive statistics, and correlations using IBM SPSS Statistics 28 software (IBM Corp., Armonk, NY, USA). It was observed that 0.4% of the data points were missing. If less than 5% of data is missing, the issue is not as significant [43]. One popular, yet conservative method for dealing with missing values is to replace them with the mean prior to the main analysis [43]. We followed this recommendation and substituted the missing values with the mean. Skew and Kurtosis analyses were used to check data normality. No extreme outliers were identified. Generally, a skewness value between −1 and +1 is considered excellent, but it is also recognized that a value between −2 and +2 is acceptable [44]. Skewness and Kurtosis for research variables did not exceed 2. For descriptive analyses, means and standard deviations were calculated for all variables. The reliability of all variables was estimated using alpha coefficients. Pearson bivariate correlations were calculated to access relations between all variables. Finally, to test hypotheses, mediation analyses were performed using the PROCESS 3.5 [45] SPSS macro (model 4). Direct and indirect effects of empowering and disempowering climates on doping likelihood were analyzed. Self-regulatory efficacy and attitudes towards doping were used as mediators. A bootstrapping method based on 5.000 samples, and bias-corrected 95% confidence intervals were calculated to assess the significance of effects. A significant effect is indicated when the confidence interval excludes zero. The completely standardized indirect effect (CSIE) served as the effect size measure, with interpretations of 0.01 indicating a small effect, 0.09 a medium effect, and 0.25 a large effect [46]. The criterion for statistical significance was defined as *p* < 0.05.

## 3. Results

### 3.1. Descriptive Statistics, Reliabilities, and Bivariate Correlations

The descriptive statistics of the variables showed that, on average, athletes perceived a high coach-created empowering climate and a lower disempowering climate (Table 1). The research data further demonstrated that athletes indicated a high level of doping self-regulatory efficacy and negative attitudes towards doping in sports. Athletes also were not likely to use doping in order to increase their performance or recovery from injuries. Based on our data, all used research measures have good internal consistency.

Concerning the relationships between the study variables, correlations showed a significant positive association between an empowering climate and doping self-regulatory efficacy, and a negative correlation with attitudes towards doping and doping likelihood. Conversely, the disempowering climate exhibited a significant positive correlation with doping attitudes and doping likelihood, while showing a negative association with doping self-regulatory efficacy.

### 3.2. Main Analyses

The primary purpose of our research was to investigate whether perceived coach-created empowering and disempowering climates predicted athletes’ doping likelihood. Additionally, we aimed to explore whether doping self-regulatory efficacy and attitudes towards doping mediated the association between perceived coach-created empowering and disempowering climates and doping likelihood.

**Figure 1:**
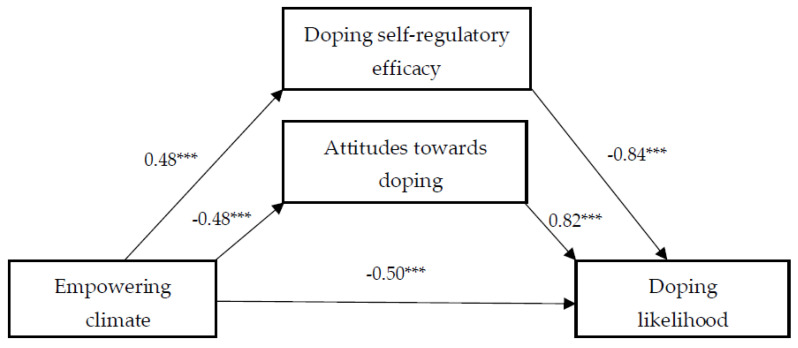
The effect of empowering climate on doping likelihood, and the mediating role of doping self-regulatory efficacy and attitudes towards doping. Note: The values provided represent the unstandardized regression coefficients. *** *p* < 0.001.

First, we examined the association between a perceived empowering climate and doping likelihood. As evident from Figure 1, a perceived empowering climate directly and negatively predicted doping likelihood and attitudes towards doping, and also positively doping self-regulatory efficacy. It was found that a perceived empowering climate had a significant indirect effect on doping likelihood via doping self-regulatory efficacy, as well as via attitudes towards doping (Table 2).

**Figure 2:**
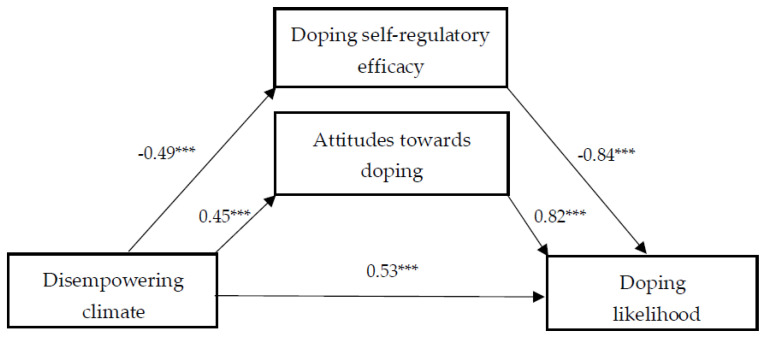
The effect of disempowering climate on doping likelihood, and the mediating role of doping self-regulatory efficacy and attitudes towards doping. Note: The values provided represent the unstandardized regression coefficients. *** *p* < 0.001.

Second, we examined the relationship between a perceived disempowering climate and doping likelihood. As can be seen in Figure 2, a perceived disempowering climate exhibited a significant positive direct influence on doping likelihood. A perceived disempowering climate is also directly positively related to attitudes and negatively to doping self-regulatory efficacy. Additionally, our findings revealed that a perceived disempowering climate had a significant indirect effect on doping likelihood via doping self-regulatory efficacy, as well as via attitudes towards doping (Table 3).

## 4. Discussion

The aim of this research was to investigate the relationship between athletes’ perceptions of coach-created empowering and disempowering motivational climates and doping likelihood, and whether the attitudes towards doping and doping self-regulatory efficacy mediates these relationships. In general, the findings were consistent with our initial hypothesis. We hypothesized that a perceived empowering motivational climate would show a negative association with doping likelihood, and that both attitudes towards doping and doping self-regulatory efficacy would mediate this relationship.

We found that a coach-created empowering motivational climate was negatively associated with doping likelihood. Accordingly, a disempowering climate had a significant positive influence on doping likelihood. Given that an empowering climate is defined as encouraging task involvement, supporting autonomy, and promoting social support, whereas a disempowering climate is best described by ego-involving and controlling characteristics [26], our study’s findings align with those of prior research. Prior studies showed that athletes who perceived their coach communicating that winning in sports is the most important thing (focused on ego-involving climate) were more likely to indicate a higher doping likelihood [31] and also doping use [8]. In contrast, a more task-oriented environment leads to more task-oriented players [47], and task orientation tends to be negatively related to doping likelihood [48]. However, in making these comparisons, it should be emphasized that the studies mentioned revealed links between the separate components of an empowering or disempowering climate. To date, only one study was conducted which aimed to analyze the relationship between athletes’ predisposition to acceptance of cheating and gamesmanship and the perception of empowering and disempowering climates, and what is more, the climate was measured with the same measures as in our study [32]. This research indicated that the perception of a disempowering climate is related to the acceptance of cheating. As Borrueco et al. [32] did not specifically focus on doping, the relationships revealed in our study between the empowering and disempowering climates created by coaches and athletes’ likelihood to dope not only partially replicated the results of study mentioned above but also complemented them.

The study’s results suggest that it is important to understand what kind of motivational climate the coach creates in the team. It should be noted that athletes and coaches have different perceptions of the motivational climate created in the team [49]. Also, coaches may want to create a more empowering climate in the team, but they lack both knowledge and pedagogical competences to realize it [50]. Moreover, a coach-created motivational climate in the team also depends on other factors, such as experience, performance level, and the requirements for coaches themselves [50]. In the context of coach anti-doping behavior, it also depends on the various factors mentioned above [51].

We also found that both empowering and disempowering climates had a significant indirect effect on doping likelihood via attitudes towards doping. Previous qualitative studies have highlighted the importance of a motivational climate in relation to attitudes towards doping [35,52,53]. The results from the studies by Hodge et al. [52] and Chen et al. [53] showed that a controlling coaching style positively correlated with attitudes towards doping. Therefore, a coach-created empowering motivational climate in sports can negatively influence athletes’ doping likelihood. However, if coaches encourage a disempowering motivational climate, it can positively affect athletes’ doping likelihood. The possible reasons may be that a coach-created empowering motivational climate encourages athletes to overcome difficulties in sports on their own by improving skills and enjoying the process in pursuit of success, which influences their attitudes towards doping and thus weakens the potential doping likelihood [35]. On the other hand, when a coach creates a more controlling and less autonomy-encouraging motivational climate, athletes perceive success in sports as surpassing opponents, supremacy over others, and victory [35]. When athletes perceive success in such a way, they are more likely to cheat in sports [31,54].

Our results showed that both empowering and disempowering climates had a significant indirect effect on doping likelihood via doping self-regulatory efficacy. Accordingly, previous research found a positive relationship between doping self-regulatory efficacy and a lower intention to dope [17]. Based on Ring and Kavussanu [15] findings, athletes that have a high level of doping self-regulatory efficacy are less prone to doping. Ntoumanis et al. [8] found that self-efficacy had a strong negative association with the intention to use illegal substances and doping behavior. When discussing why an empowering climate created by a coach could affect athletes’ intentions to use doping via self-regulatory efficacy, it ought to be remembered that a coach may improve athletes’ confidence in their ability to resist pressure to use doping by boosting athletes’ autonomy, providing social support, and promoting individual progress. Therefore, athletes gain more confidence in themselves and believe in their ability to resist doping.

In summary, this study’s results suggest that by creating an empowering motivational climate, coaches provide beneficial help for the athletes, because they encourage athletes to believe in their abilities to resist doping and develop more negative attitudes towards doping, and thus it inversely effects athletes’ doping likelihood in sports. In contrast, the disempowering climate not only harms the belief in an athlete’s own ability to resist doping—hence, encouraging athletes to dope—but also develops more positive attitudes towards doping and, as a consequence, influences doping likelihood. The results of our study indicate that interventions created to increase an empowering motivational climate could be effective, at least in part, by increasing athletes’ doping self-regulatory efficacy and developing athletes’ negative attitudes towards doping.

*Limitation and future research directions*: Our study revealed interesting findings. However, it is important to note some limitations of the study which may be important in interpreting these results. First, we did not study athletes’ experiences of doping or actual doping behavior. Therefore, based on our data, it would be incorrect to make conclusions about to what extent the disempowering coach-created climate is associated with the use of doping. Second, athletes’ doping intentions were investigated using only two hypothetical situations. Some authors [42] recommend using a greater variety of situations in the research of athletes’ intentions to use doping, which should be considered in further research.

Additionally, we suggest exploring several interesting issues that would be worth investigating in the future research. High-performance sports are highly competitive, and athletes may feel pressure to perform well. Nonetheless, such pressure can instill in athletes a fear of making mistakes and a fear of failure [55], which is associated with antisocial behavior [56,57]. A recent study discovered that a perceived empowering motivational climate is directly negatively associated with the fear of failure, while a disempowering one has the opposite effect [58]. Although some studies revealed a positive relationship between the fear of failure and doping susceptibility [59], future research should examine how coach-created empowering and disempowering motivational climates are related to athletes’ fear of failure and doping behavior.

A majority of the studies on coach-created empowering and disempowering motivational climates have applied quantitative designs with athletes; therefore, it is suggested to shift the focus towards qualitative studies, which delve into athletes’ perceptions of the motivational climate [27]. We also encourage researchers to consider qualitative studies with coaches. Essentially, qualitative studies on how coaches perceive and experience the motivational climate are not entirely unexplored [60,61]. However, future studies must focus on how coaches themselves perceive the created motivational climate regarding doping and anti-doping. As coaches sometimes are lacking knowledge and competence in how to deal with doping-related issues [51], such studies expand our understanding of how a coach-created motivational climate is related to athletes’ attitudes, self-efficacy, and doping behavior. Furthermore, such studies could also be useful in the development of intervention programs aiming to help coaches to create a more empowering climate in teams and promoting anti-doping behavior. The research findings [62] suggest that such initiatives can be beneficial for both coaches and athletes.

## 5. Conclusions

This study showed novel findings on the significance of the coach-created environment, including both empowering and disempowering climates, in predicting athletes’ intentions to use doping. Our findings indicate that athletes who perceive a more empowering motivational climate created by their coach are less prone to use illegal substances, and this relationship is mediated by the attitudes towards doping and doping self-regulatory efficacy. On the contrary, a disempowering climate is positively related to athletes’ doping likelihood. However, as a cross-sectional study design was used, replicating our results using longitudinal or experimental research designs would be important, as they could offer clearer evidence regarding the causal directions.

## Figures and Tables

**Table 1:** Descriptive statistics, alpha coefficients, and correlations.

Variables	A	M	SD	1	2	3	4
1. Empowering climate	0.93	4.13	0.62				
2. Disempowering climate	0.93	2.58	0.84	−0.57 **			
3. Doping self-regulatory efficacy	0.97	5.89	1.35	0.48 **	−0.49 **		
4. Doping attitudes	0.94	1.67	0.78	−0.48 **	0.45 **	−0.74 **	
5. Doping likelihood	0.92	1.98	1.30	−0.52 **	0.53 **	−0.84 **	0.82 **

Note: ** *p* < 0.01.

**Table 2:** Indirect effects of empowering climate on doping likelihood through self-regulatory efficacy and attitudes towards doping.

Relationship	Indirect Effect	Confidence Interval	
		LL 95% CI	UL 95% CI
Empowering climate → doping self-regulatory efficacy → doping likelihood	−0.40 *	−0.43	−0.34
Empowering climate → attitudes → doping likelihood	−0.39 *	−0.43	−0.31

Note: * *p* < 0.05, LL95% CI = lower limit of 95% confidence interval, UL95% CI = upper limit of 95% confidence interval.

**Table 3:** Indirect effects of disempowering climate on doping likelihood through self-regulatory efficacy and attitudes towards doping.

Relationship	Indirect Effect	Confidence Interval	
		LL 95% CI	UL 95% CI
Disempowering climate → doping self-regulatory efficacy → doping likelihood	0.41 *	0.18	0.27
Disempowering climate → attitudes → doping likelihood	0.37 *	0.16	0.24

Note: * *p* < 0.05, LL95% CI = lower limit of 95% confidence interval, UL95% CI = upper limit of 95% confidence interval.

## Data Availability

The data that support the findings of this study are available from the corresponding author (B.H.) upon reasonable request.
